# Transcriptome Analysis of the Preterm Rabbit Lung after Seven Days of Hyperoxic Exposure

**DOI:** 10.1371/journal.pone.0136569

**Published:** 2015-08-28

**Authors:** Thomas Salaets, Jute Richter, Paul Brady, Julio Jimenez, Taro Nagatomo, Jan Deprest, Jaan Toelen

**Affiliations:** 1 University Hospitals Leuven, Department of Pediatrics, Leuven, Belgium; 2 Department of Development and Regeneration, Research Unit Fetus Placenta Neonate, Group of Biomedical Sciences, KU Leuven, Leuven, Belgium; 3 University Hospitals Leuven, Department of Obstetrics and Gynecology, Leuven, Belgium; 4 Department of Genetics, Group of Biomedical Sciences, KU Leuven, Leuven, Belgium; 5 Clínica Alemana, Departamento Ginecología y Obstetricia, Santiago, Chile; 6 Ehime Prefectural Central Hospital, Department of Neonatology, Matsuyama, Japan; The Ohio State Unversity, UNITED STATES

## Abstract

The neonatal management of preterm born infants often results in damage to the developing lung and subsequent morbidity, referred to as bronchopulmonary dysplasia (BPD). Animal models may help in understanding the molecular processes involved in this condition and define therapeutic targets. Our goal was to identify molecular pathways using the earlier described preterm rabbit model of hyperoxia induced lung-injury. Transcriptome analysis by mRNA-sequencing was performed on lungs from preterm rabbit pups born at day 28 of gestation (term: 31 days) and kept in hyperoxia (95% O_2_) for 7 days. Controls were preterm pups kept in normoxia. Transcriptomic data were analyzed using Array Studio and Ingenuity Pathway Analysis (IPA), in order to identify the central molecules responsible for the observed transcriptional changes. We detected 2217 significantly dysregulated transcripts following hyperoxia, of which 90% could be identified. Major pathophysiological dysregulations were found in inflammation, lung development, vascular development and reactive oxygen species (ROS) metabolism. To conclude, amongst the many dysregulated transcripts, major changes were found in the inflammatory, oxidative stress and lung developmental pathways. This information may be used for the generation of new treatment hypotheses for hyperoxia-induced lung injury and BPD.

## Introduction

Preterm birth leads to a dysregulated development in many organs that are not yet adapted to postnatal life. In the pulmonary system, this often results in bronchopulmonary dysplasia (BPD), a complex disease in which multiple factors interact. Premature lungs (most frequently in the saccular stage of lung development) are exposed to hyperoxic and hyperbaric conditions during ventilation and administration of supplementary oxygen. This process is often amplified by pre- or postnatal infections, fluid imbalance, malnutrition, genetic predispositions, etc. Persistent inflammation overwhelms natural tissue repair and leads to an arrest in alveolar development and vasculogenesis.[1] The resulting lung parenchyma is composed of rudimentary alveoli, interstitial thickening and a dysmorphic capillary configuration.[2] These morphological changes can be categorized as a developmental arrest.

The aforementioned risk factors are well-known and the application of less invasive ventilation strategies and permissive hypoxemia have proven their efficacy. They became the cornerstones of current neonatal management. [3,4] Despite this, BPD continues to be a frequent complication of premature birth. About 15–25% of very low birth weight (VLBW) infants develop BPD, and rates in extremely low birth weight (ELBW) infants are even higher.[5] Moreover, BPD remains an important risk factor for lung disease later in life.[6]

At the molecular level BPD is still poorly studied compared to other pathologies. Several individual molecules assessed in bronchoalveolar fluid have been proposed to play a key role in BPD like interleukin-8 or matrix metalloproteinase-3.[7,8] Most studies however where hypothesis driven or they examined certain pathways in lung disease and repair.

Animal models are needed to study disease mechanisms and to evaluate new preventive or therapeutic strategies for BPD. Most research into BPD has been performed in hyperoxia-exposed rodent models.[9–11] Unfortunately, their lung development differs from humans as birth occurs in the early saccular stage of lung development. In rodents, alveolization only starts several days postnatally while humans start alveolizing in utero. This entails that not all the relevant findings can be extrapolated to the human context. As such, there is an advantage to study the hyperoxia induced lung injury in animal models that mimic human development more closely. The rabbit is considered a large animal model that has favorable characteristics for this research question. In contrast to rodents, rabbits indeed start alveolizing prior to birth, as do pigs, sheep, primates and humans.[12] Moreover, rabbits are easy to handle and house, have a large litter size and allow technical manipulation of the fetus at relevant developmental stages which makes them ideal for the study of effects of perinatal interventions. Therefore we used the rabbit as a model for the study of hyperoxia-induced lung injury in the preterm born pup.[13]

Rather than focus on single putative molecule or pathways, we herein used a more complete ‘systems biology’-approach,[14] that allows the exploration of larger patterns and networks. Herein we analyze transcriptome data using software (IPA) that combines expression data with current generic knowledge of molecular interactions. Our goal was to obtain more complete insights in the pathophysiology and generate new theoretical therapeutic strategies for preterm hyperoxia induced lung injury.

## Materials and Methods

### Animal model

We earlier described in detail the preterm rabbit model for hyperoxia-induced lung injury.[13] Briefly, time mated pregnant does underwent cesarean section at 28 days (term = 31 days) of gestation (early saccular lung developmental phase). The pups were randomly divided into two groups: (1) the normoxia group, where pups were housed in 21% oxygen and (2) the hyperoxia group, where pups were nursed in hyperoxia (≥ 95% oxygen); both for seven days. The model has been earlier described in detail elsewhere [15–17]. Briefly, immediately after delivery, pups were placed in an incubator at 32°C, fed twice daily via an orogastric tube and received prophylactic antibiotics and vitamin K. All animals were treated according to current guidelines of animal well-being, and the experiments were approved by the Ethics Committee for Animal Experimentation of the Faculty of Medicine of the Katholieke Universiteit Leuven (Leuven, Belgium).

### Harvesting of specimens

At day 7 of life, pups were harvested for histological and transcriptome analysis. After euthanizing the pups with a mixture of embutramide 200mg, mebezonium 50mg and tetracain hydrochloride 5mg (intracardiac injection of 0.1mL T61, Intervet Belgium NV, Mechelen, Belgium), thoracotomy was performed and both lungs and trachea were removed “en bloc”. The left bronchus was ligated and the left lung was snap-frozen, while the right lung was processed for histological analysis (see below). Six pups per group were used for histological analysis. Out of the snap frozen samples, four left lungs from the normoxia group, and four from the hyperoxia group were randomly selected for transcriptome analysis.

### Lung injury score

A 20G catheter was inserted in the trachea where after the right lung was fixed with 4% paraformaldehyde by immersion and perfusion under a constant hydrostatic pressure of 25cm H_2_O for 24 hours before embedding. Paraffin sections were stained with hematoxylin and eosin (HE) for further histological analysis. Lung injury score was assessed on 20 random high-power fields (400x total magnification) for each pup. The selection of random fields was obtained by successive random displacements (each at least one high power field in length) from the initial position provided that at least 50% of each field was occupied by lung alveoli. A three-tiered scheme was used to quantify each of five histological parameters: presence of neutrophils in alveolar and interstitial space, hyaline membranes, debris filling the airspaces and septal thickening.[18] The data were analyzed statistically using student-T test, significance was set with a p-value < 0.05.

### RNA isolation and sequencing

RNA isolation was performed on snap frozen lung tissue using the RNeasy mini kit (Qiagen). Tissue lysis and homogenization was performed in 1200μL Buffer RLT using the TissueLyser system (Qiagen). Following tissue disruption and homogenization, samples were centrifuged for 3 min at 14000rpm in a benchtop micro centrifuge. Lysate was transferred to fresh tubes and an equal volume of 70% ethanol was added. 600μL of sample was added twice to a spin column, with 2 RNeasy spin columns used per sample. Following wash steps RNA was eluted in 50μL RNAse-free H2O. Total RNA quantification was performed using the Nanodrop 1000 spectrophotometer (Thermo Scientific). RNA integrity was assessed using the RNA 6000 Nano Kit and the Bioanalyser (Agilent Technologies) according to the manufacturer’s recommendations. Between 1 and 2μg of total RNA was used as input material for sequencing library preparation which was performed with the TruSeq RNA Library Preparation Kit (Illumina) according to the manufacturers protocol. Fragmentation was performed for 6 minutes. 8 PCR cycles were used for PCR enrichment step. Samples were indexed to allow for multiplexing. Sequencing libraries were quantified using the Qubit fluorometer (Life Technologies). Library quality and size range was assessed using the Bioanalyser (Agilent Technologies) with the DNA 1000 Kit (Agilent Technologies) according to the manufacturer’s recommendations. Individual libraries were diluted to a final concentration of 2nM and pooled for sequencing. Pooled libraries were then sequenced in a single lane of an Illumina HiSeq2000 flow cell generating single end 50bp reads.

### Data analysis

Expression values were calculated per gene and normalized to reads per kilobase per million reads (RPKM) values as described by Mortazavi.[19] An RPKM value of >1 was applied as cut-off to distinguish between ‘noisy’ or ‘leaky’ transcription and ‘true’ mRNA expression levels for comparison between samples. For comparison between both groups, fold changes (FC) were calculated, indicating the ratio of expression. We applied a cut-off of >2 or <-2, in order to decrease the number of transcripts in our analysis. False discovery rates (FDR) were calculated as a measure for statistical significance of the difference in expression of a gene between the two groups. We considered a transcript change significant if FDR was <0.01.

Further analysis was performed using the Ingenuity Pathway Analysis (IPA) software. The IPA ‘Upstream Regulator Analysis’ predicts upstream regulators by combining the directional expression changes from our mRNA-sequencing, and knowledge from prior experimental reports on causal effects between molecules (endogenous and exogenous), compiled in the IPA Knowledge Base. Upstream Regulator Analysis calculates a z-score based on the edge of dysregulation of all the downstream molecules and the uniformity of the existing evidence about the upstream-downstream relation, for every upstream regulator known to have a causal effect on at least 4 dysregulated transcripts. Z-scores of <-2 and >2 respectively indicate a significant inhibition and activation state of the upstream regulator, regardless of the actual expression level of these molecules.[20] The Network Generation Algorithm links molecules based on experimentally observed interactions, and orders these molecule based on their interconnectedness. In general, the more interactions with other network members, the more central a molecule will be in a network.

## Results

### Lung injury score

**Fig 1:**
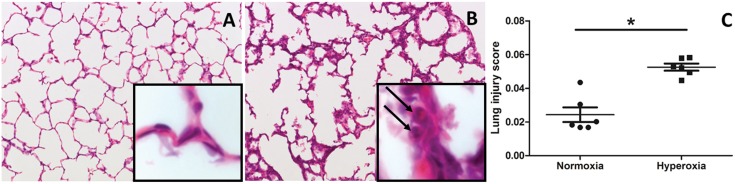
HE staining and lung injury score. A-B: HE staining of a pressure fixed lung of pups held in normoxia (A) or hyperoxia (B) for 7 days of life. In the magnification square, neutrophils are marked by the arrows. C: lung injury score assessing 5 different parameters: neutrophils in alveolar airspace, neutrophils in the interstitial space, hyaline membranes, proteinaceous debris filling the airspace, alveolar septal thickening.

Histological assessment of both normoxia as well as hyperoxia exposed lungs reveals a clear increase in neutrophil infiltration and alveolar wall thickening (Fig 1A and 1B).[13] Generating a combined lung injury score revealed a significant difference between both groups (Fig 1C) with an increase in lung injury observed in animals held in hyperoxia (p<0.01).

### Hyperoxia and gene expression profiles

**Fig 2:**
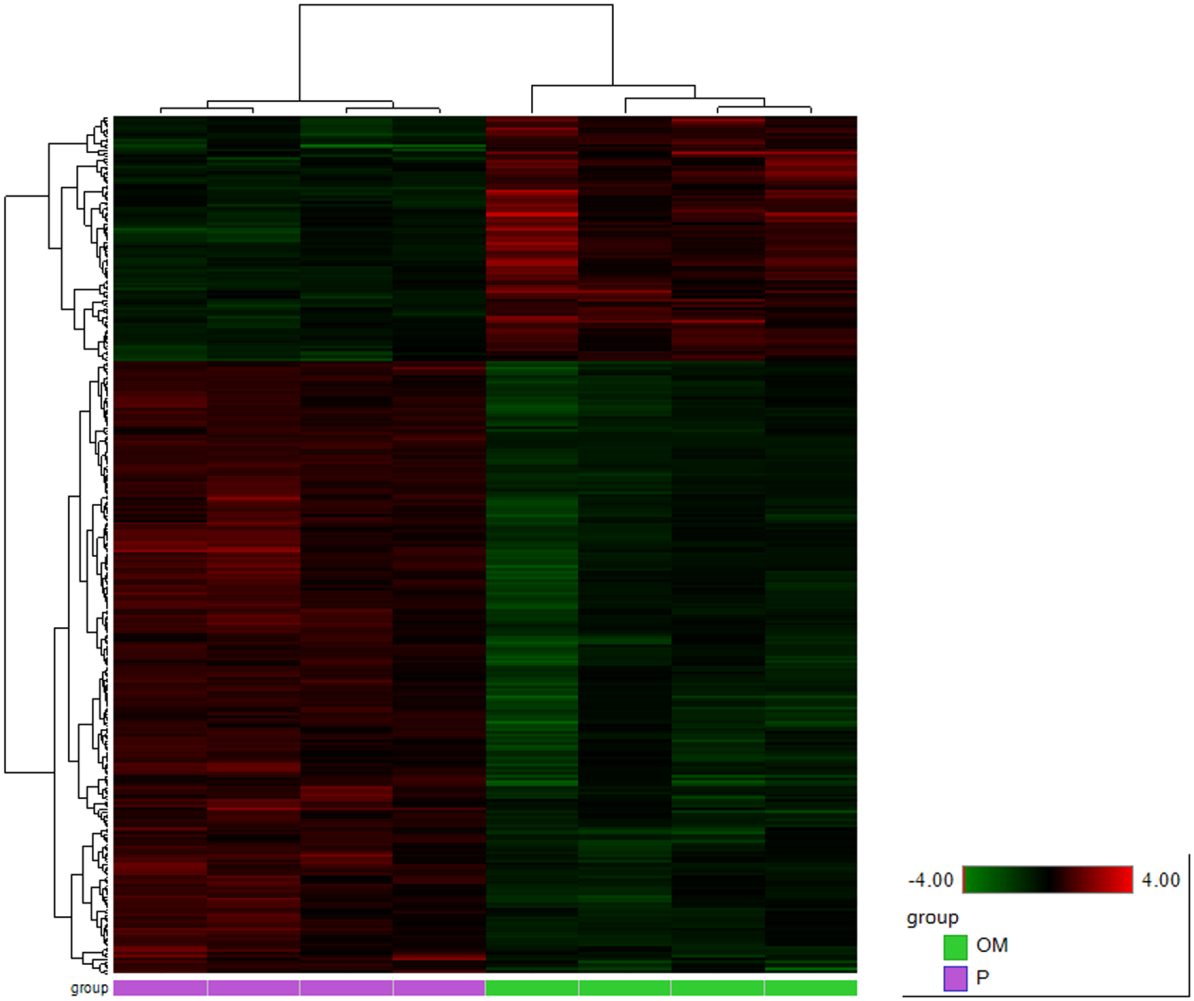
Heat map of differentially expressed genes. Animals held in normoxia (to the left, purple) or in hyperoxia (to the right, green) with FC+-2 and FDR <0.01. Color intensity displayed in the heat map are the Log2 transformed RPKM gene expression value. These are normalized to relative low (green) and high (red) signal intensities shown in the heat map key.

We identified 2217 transcripts that were dysregulated (FDR < 0.01) in hyperoxia by at least a factor 2 (FC>2 or FC<-2). A heat map of these genes visualizes a clear distinction between hyperoxia and normoxia. (Fig 2). We were able to link IPA-gene names to 1989 of the 2217 dysregulated transcripts. This group was used for further analysis in IPA. 1023 of these transcripts are upregulated in hyperoxia, with fold changes up to +235.786. The other 966 are downregulated, as far down as -38.476. The ten most upregulated molecules in hyperoxia are shown in Table 1, the ten most downregulated in Table 2. A complete list of these 1989 dysregulated molecules can be found in S1 Table.

**Table 1:** Upregulated molecules. Ten most upregulated molecules in our dataset; FC: fold change, FDR: false discovery rate.

Rank	IPA name	Full name	FC	FDR
**1**	RLN1	Relaxin 1	235.786	1.04x10^-5^
**2**	MT-ND4L	NADH dehydrogenase subunit 4L	153.771	6.80x10^-3^
**3**	CCL2	Chemokine (C-C motif) ligand 2	148.355	1.20x10^-5^
**4**	MMP3	Matrix metalloproteinase 3	100.723	7.57x10^-5^
**5**	BIRC 3	Baculoviral IAP repeat containing 3	53.728	2.48x10^-5^
**6**	PTX3	Pentraxin 3, long	43.956	4.00x10^-4^
**7**	SRXN1	Sulfiredoxin 1	42.437	2.00x10^-5^
**8**	CXCL8	Chemokine (C-X-C motif) ligand 8	37.831	2.63x10^-5^
**9**	DKK1	Dickkopf WNT signaling pathway inhibitor 1	34.030	7.57x10^-5^
**10**	LIF	Leukemia inhibitory factor 1	33.378	6.00x10^-4^

**Table 2:** Down regulated molecules. Ten most down regulated molecules in our dataset; FC: fold change, FDR: false discovery rate.

Rank	IPA abbreviation	Name	FC	FDR
**1**	VIPR1	Vasoactive intestinal peptide receptor 1	-38.476	2.69x10^-5^
**2**	C2orf40	Chromosome 2 open reading frame 40	-32.645	1.00x10^-4^
**3**	ACE	Angiotensin I converting enzyme	-26.315	8.19x10^-6^
**4**	CD300LG	CD300 molecule-like family, member g	-22.293	1.39x10^-5^
**5**	SCBG3A1	Secretoglobulin, family 3A, member 1	-21.677	8.40x10^-3^
**6**	S100A1	S100 calcium binding protein A1	-19.873	3.59x10^-5^
**7**	CYP1A1	Cytochrome 450, family 1, subfamily A, polypeptide 1	-17.727	2.00x10^-4^
**8**	COLEC10	Collectin subfamily member 10 (C-type lectin)	-15.835	2.00x10^-4^
**9**	CA4	Carbonic anhydrase IV	-14.724	5.00x10^-4^
**10**	APLNR	Apelin receptor	-14.407	4.38x10^-5^

### Pathway analysis of differentially expressed genes

Running the Upstream Regulator Analysis, we identified 58 inhibited and 160 activated significant upstream regulators. Both endogenous and exogenous molecules are included in this analysis. All significant upstream regulators which are classified as transcription factors are displayed in Table 3. All other upstream regulator types can be found in S2 Table. Significantly dysregulated molecules were sorted by their function or involvement in pathophysiological processes. An overview of the most relevant dysregulated molecules is provided in Table 4.

**Table 3:** Upstream regulators. Transcription regulators, recognized to be upstream regulators (z-score +-2), with actual fold change, z-score, p-value of overlap and number of downstream effectors.

IPA ID	Entrez Gene Name	FC	Z-score	p-value of overlap	# of DTM
Activated upstream transcription factors					
NFE2L2	nuclear factor, erythroid 2-like 2		4.796	3.80x10^-13^	76
CREB1	cAMP responsive element binding protein 1		3.661	5.10x10^-8^	79
RELA	v-rel avian reticuloendotheliosis viral oncogene homolog A		2.998	1.25x10^-5^	49
NFKB1	nuclear factor of kappa light polypeptide gene enhancer in B-cells 1		2.952	2.15x10^-3^	29
CDKN2A	cyclin-dependent kinase inhibitor 2A		2.808	1.67x10^-5^	33
ATF4	Activating transcription factor 4	2.637	2.490	1.59x10^-14^	38
SMAD2	SMAD family member 2		2.425	1.00	6
KDM5B	lysine (K)-specific demethylase 5B		2.400	4.19x10^-4^	21
MYC	v-myc avian myelocytomatosis viral oncogene homolog	3.143	2.359	1.59x10^-12^	136
HMGB1	high mobility group box 1		2.303	6.46x10^-2^	10
MAFK	v-maf avian musculoaponeurotic fibrosarcoma oncogene homolog K		2.170	1.00x10^-2^	5
EHF	ets homologous factor		2.121	1.35x10^-1^	9
MAFG	v-maf avian musculoaponeurotic fibrosarcoma oncogene homolog G		2.000	7.58x10^-3^	5
MAFF	v-maf avian musculoaponeurotic fibrosarcoma oncogene homolog F		2.000	9.41x10^-3^	4
Inhibited upstream transcription factors					
TWIST1	twist family bHLH transcription factor 1		-3.182	8.83x10^-8^	32
GATA2	GATA binding protein 2		-2.724	3.03x10^-6^	19
KLF2	Kruppel-like factor 2		-2.396	1.42x10^-6^	28
PRDM1	PR domain containing 1, with ZNF domain		-2.255	5.26x10^-3^	17
NEUROG3	neurogenin 3		-2.219	1.00	5
HOXA7	homeobox A7		-2.200	8.52x10^-2^	5
HOXA9	homeobox A9		-2.183	1.74x10^-1^	18
PLAG1	pleiomorphic adenoma gene 1		-2.164	4.62x10^-3^	11
GLI2	GLI family zinc finger 2		-2.159	6.27x10^-2^	11
NEUROG1	neurogenin 1		-2.111	1.63x10^-2^	11

DTM: dysregulated target molecules

**Table 4:** Dysregulated genes of special interest. Dysregulated genes of special interest for hyperoxia-induced lung injury; in order of appearance; FC: fold change, FDR: false discovery rate.

IPA ID	Full Name	FC	FDR
Inflammation			
IL1A	Interleukin 1, alpha	6.864	3.60x10^-3^
IL1B	Interleukin 1, beta	5.092	3.70x10^-3^
PTGS2	Prostaglandin-endoperoxide synthase 2	23.625	7.00x10^-4^
NOS2	Nitric oxide synthase 2, inducible	-8.495	1.90x10^-5^
CSF3	Colony stimulating factor 3 (granulocyte)	17.090	2.30x10^-3^
CCL2	Chemokine (C-C motif) ligand 2	148.355	1.20x10^-5^
CXCL8	Chemokine (C-X-C motif) ligand 8	37.831	2.63x10^-5^
Lung development			
PPARG	Peroxisome proliferator-activated receptor gamma	4.268	2.90x10^-3^
SPP1	Secreted phosphoprotein 1	28.159	3.30x10^-3^
CAV1	Caveolin 1, caveolae protein, 22kDa	-6.282	3.10x10^-5^
DKK1	Dickkopf WNT signaling pathway inhibitor 1	34.030	7.57x10^-5^
ACE	Angiotensin I converting enzyme	-26.315	8.19x10^-6^
Vasculogenesis			
VEGFA	Vascular endothelial growth factor A	-5.539	1.00x10^-4^
TEK	TEK tyrosine kinase, endothelial	-7.844	1.90x10^-5^
TIE1	Tyrosine kinase with immunoglobulin-like and EGF-like domains 1	-6.540	1.82x10^-5^
ANGPT2	Angiopoetin 2	12.089	7.00x10^-4^
ROS metabolism			
SOD2	Superoxide dismutase 2, mitochondrial	2.349	8.90x10^-3^
TXNRD1	Thioredoxin reductase 1	7.262	1.20x10^-3^
SRXN1	Sulfiredoxin 1	42.437	2.00x10^-5^
CYP1A1	Cytochrome 450, family 1, subfamily A, polypeptide 1	-17.727	2.00x10^-4^

#### Inflammation

**Fig 3:**
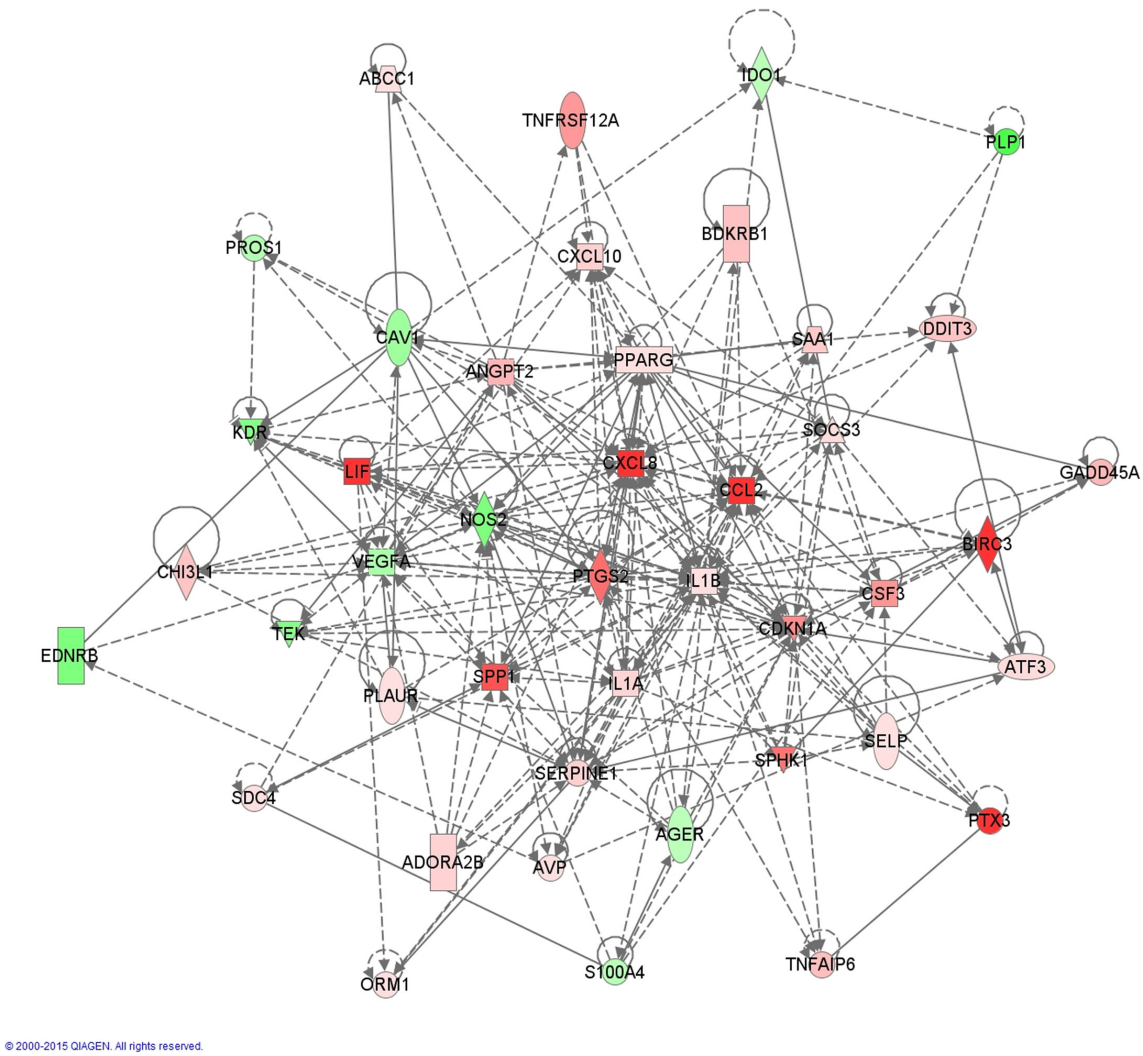
Network of dysregulated molecules involved in IPA-function: ‘Inflammatory Response’. The following molecules play a central role: IL1A (FC 6.864), IL1B (FC 5.092), PTSG2 (FC 23.625), NOS2 (FC -8.495), CXCL8 (FC 37.831) and CCL2 (FC 148.355). In order to increase readability, only molecules with a FC of +/- 4 and 3 or more connections are shown in this figure.

Although the inflammatory mediators are numerous, several appear to be active molecules, such as interleukin 1A (IL1A, FC 6.864, FDR 3.6x10^-3^), interleukin 1B (IL1B, FC 5.092, FDR 3.7x10^-3^), cyclo-oxygenase 2 (PTGS2 or COX2, FC 23.625, FDR 7.00x10^-4^), nitric oxide synthase 2 (NOS2, FC -8.495, FDR 1.90x10^-5^), granulocyte colony stimulating factor (G-CSF or CSF3, FC 17.090, FDR 2.30x10^-3^) and chemokines (C-C motif) ligand 2 (CCL2, FC 148.355, FDR 1.20x10^-5^) and (C-X-C motif) ligand 8 (CXCL8 or IL8, FC 37.831, FDR 2.63x10^-5^).(Fig 3) Upstream analysis highlights tumor necrosis factor-alfa (TNFα) as a major regulator (z-score 4.465). Downstream effectors of the TNFα-pathway include inflammatory mediators (IL1A, IL1B) but also molecules involved in vasculogenesis (vascular endothelial growth factor A (VEGFA), angiopoietin 2 (ANGPT2)), lung development (secreted phosphoprotein 1 (SPP1), caveolin 1 (CAV1), dickkopf WNT signaling pathway inhibitor 1 (DKK1), peroxisome proliferator-activated receptor γ (PPARγ)) and ROS metabolism (cytochrome P450 1A1 (CYP1A1), superoxide dismutase 2 (SOD2)) (S3 Table). Nuclear factor of kappa light polypeptide gene enhancer in B-cells 1 (NF-κB, z-score 2.952), the intracellular transcriptional effector of TNFα is also recognized as an activated upstream regulator. Other significantly dysregulated inflammatory upstream regulators are interferon γ (IFNγ) and several other interleukins (1A, 1B, 2, 3, 4, 5, 6, 12B, 17A, 17F, 18) albeit with lower z-scores (2.756, 3.269, 4.525, 2.004, 3.235, 2.247, 2.131, 2.369, 2.049, 2.658, 2.574, 2.348 resp.). The central molecules involved in the inflammatory pathway have been displayed in Fig 3.

#### Lung development

**Fig 4:**
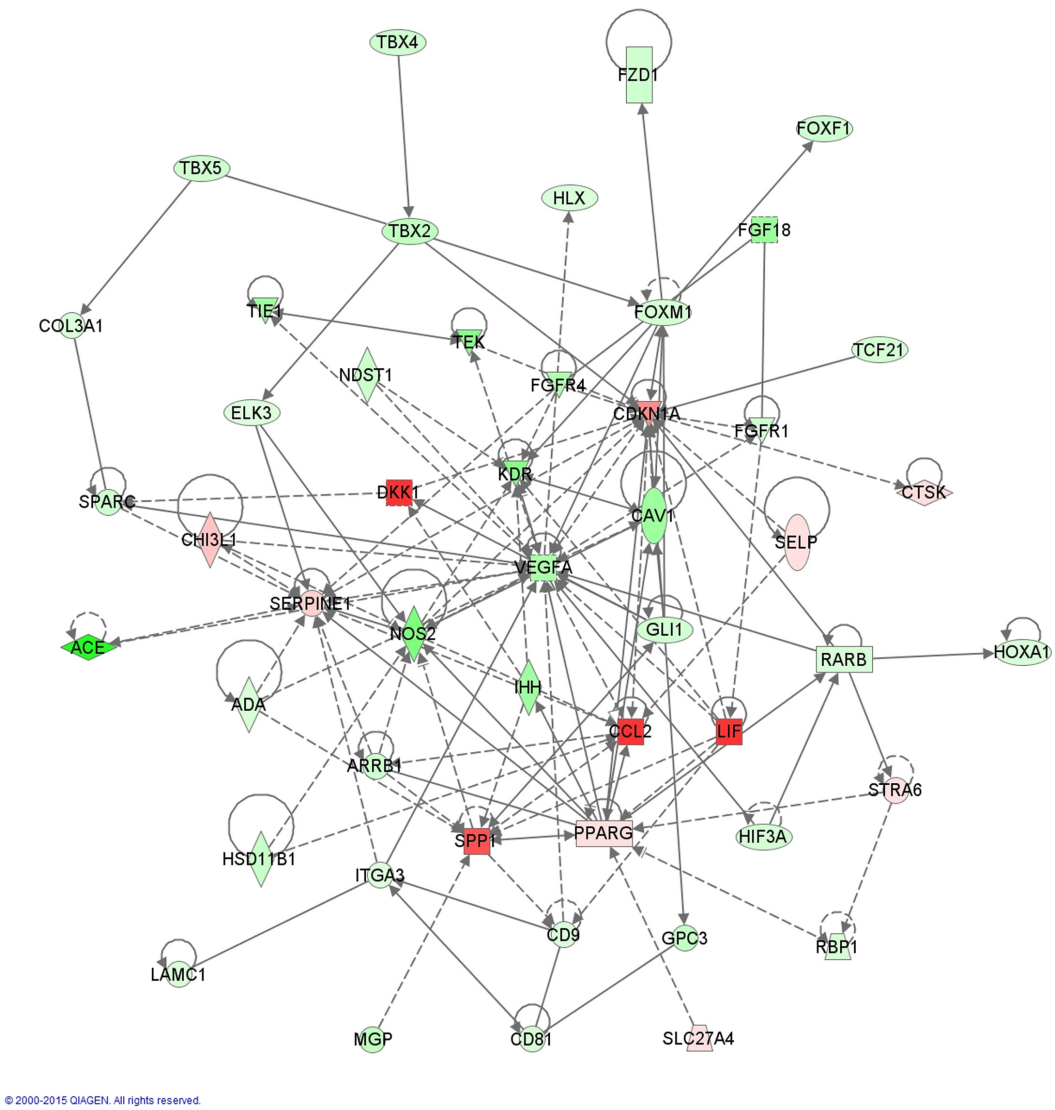
Network of dysregulated molecules involved in IPA-function ‘Formation of lung’. Central, broadly connected molecules are SPP1 (FC 28.159), CAV1 (FC -6.282), PPARγ (FC 4.268), NOS2 (FC -8.495) and VEGFA (FC 5.539). Relevant mediators of lung development mentioned in the following references are added [26,27,30,34].

Molecules involved in lung development which are dysregulated in our model are shown in Fig 4. Central molecules in this network are PPARγ (FC 4.268, FDR 2.90x10^-3^), SPP1 or osteopontin (FC 28.159, FDR 3.30x10^-3^), CAV1 (FC -6.282, FDR 3.10x10^-5^), NOS2 and VEGFA. Other highly dysregulated molecules in this network are DKK1 (FC 34.030, FDR 7.57x10^-5^) and angiotensin I converting enzyme (ACE, FC -26.315, FDR 8.19x10^-6^). Our upstream analysis identified KLF2 (z-score -2.396) as an important inhibited transcription factor. Downstream effectors of KLF2 include mediators of inflammation (CCL2, CXCL8, PTGS2), vasculogenesis (ANGPT2) and lung development (PPARγ, ACE).(S4 Table)

#### Vasculogenesis

VEGFA (FC -5.539, FDR 1.00x10^-4^) is down regulated in our model. TEK tyrosine kinase (TEK or TIE2, FC -7.844, FDR 1.90x10^-5^) and tyrosine kinase with immunoglobulin-like and EGF-like domains 1 (TIE1, FC -6.540, FDR 1.82x10^-5^), the molecular receptors of the angiopoetins, are down regulated while angiopoetin 2 (ANGPT2, FC 12.089, FDR 7.00x10^-4^, z-score 2.612), a receptor antagonist, is upregulated and also recognized as a significantly activated upstream regulator.

#### ROS metabolism

Several enzymes involved in scavenging and detoxifying the reactive oxygen species are upregulated, like superoxide dismutase 2 (SOD2, FC 2.349, FDR 8.90x10^-3^), thioredoxin reductase 1 (TXNRD1, FC 7.262, FDR 1.20x10^-3^) and sulfiredoxin 1 (SRXN1, FC 42.437, FDR 2.00x10^-5^). Furthermore several cytochrome P450 (CYP) enzymes are down regulated, especially CYP1A1 (FC -17.727, FDR 2.00x10^-4^). A major upstream regulator influencing the above mentioned enzymes is nuclear factor erythroid 2-like 2 (NFE2L2, z-score 4.796), downstream effectors include inflammatory mediators (IL1B, PTSG2, …) as well as VEGFA (S5 Table).

## Discussion

Under the assumption that a rabbit model has several advantages over rodent models for studying BPD with regard to lung development and the potential of experimental therapeutic interventions, we used this preterm rabbit model [13] to comprehensively describe possible pathways involved in the pathophysiology of BPD using modern techniques. Herein, we used whole transcriptome analysis as this offers an comprehensive amount of expression information, that allows to document the intricate and complex processes that occur in the damaged developing lung. Lung injury score demonstrated results that parallel lung damage seen in clinical BPD-samples.[2] This confirms the relevance of insights in the molecular mechanisms of BPD obtained in our model. We focused on four relevant biological networks in this context: inflammation, lung development, vasculogenesis and ROS metabolism.

Several inflammatory mediators were significantly dysregulated in our model, confirming an important role for inflammation in the pathogenesis of hyperoxia-induced lung injury. The pattern of dysregulation of these molecules is consistent with a pro-inflammatory state. This is in line with data from other animal models [21] and clinical studies.[22] The increased TNFα-activity induces many of the transcriptional changes seen in our model, some of these downstream mediators have been described in hyperoxia exposed term rabbit lungs.[23] It remains unclear if this TNFα-activity is increased as a protective pathway or merely secondary to the inflammation in the preterm lung. Experimental therapy both with TNFα and anti-TNFα show an improvement in rats exposed to hyperoxia.[24,25] To date this has not been corroborated in larger animal models.

Many preclinical studies have reported on molecules involved in lung development [26–29] that can account for the developmental arrest in BPD. Several of these lung development molecules are also dysregulated in our model.(Fig 4) PPARγ, which was also an upregulated molecule in our study, is important for normal lung maturation.[30] Furthermore, beneficial effects have been achieved with stimulation of PPARγ, e.g. by rosiglitazone in the rat model of hyperoxia-induced lung injury.[31–33] A second upregulated molecule is DKK1, which is an inhibitor of WNT-signaling. Its known involvement in branching morphogenesis and alveolization could account for a role in the developmental arrest of BPD.[28,34] Other dysregulated factors of pulmonary development include SPP1 (downregulated during secondary septation [27]), CAV1(involved in alveolar septation [35] and acute lung injury [36]) and ACE (involved in secondary septation [37]).

We also suggest a role for KLF2, which we identified as an important upstream regulator. It is highly expressed in adult mouse lung tissue and was demonstrated to be important for late stage lung development (saccular and alveolar phase).[38] Furthermore, KLF2 is involved in endothelial homeostasis and angiogenesis [39] and has negative regulatory effects on immune cell activation.[40]

Normal lung development depends, certainly in the later stages, on vasculogenesis and the development of mesenchymal structures: blood vessels and respiratory epithelium are in constant interaction. Several molecular factors responsible for angiogenesis in the lung [26] are changed in our hyperoxia model. VEGFA seems a central molecule in most of our generated networks. The administration of VEGFA to attenuate lung damage has been tried in different models with varying results.[41,42] Furthermore knockdown of VEGF abolishes the protective effects of mesenchymal stem cells in a rat model of hyperoxic lung injury thereby demonstrating the critical role of VEGF.[43]

The upregulation of several enzymes involved in scavenging and detoxifying of ROS can be considered as protective against the potential adverse effects of hyperoxia. NFE2L2 plays a critical role in the induction of this response.[44] Another important player we identified, is CYP1A1. Induction of CYP1A1 via the aryl hydrocarbon receptor (AHR) pathway, using β-naphthoflavone or omeprazole attenuates hyperoxic lung injury, which suggests a protective effect of CYP1A1.[45,46]

We acknowledge certain limitations of our study. First, in this model we use only hyperoxia to create lung injury. This is an imperfect approximation for human ‘new’ BPD.[1,47] In human BPD, several other factors (barotrauma, infections, malnutrition, fluid imbalance, antenatal corticosteroids, surfactant, …) are involved, yet are not included in this experimental model. Secondly, there are some methodological limitations. mRNA sequencing was performed on whole, mixed, lung tissue which offers an overview of expression changes in all pulmonary cell types. This results in loss of ‘spatial resolution’ as specific up regulation in individual cell types constituting the airways, vasculature and parenchyma cannot be quantified in this manner. Furthermore, our samples were only examined at a single time point (7 days of hyperoxic exposure), a loss of temporal resolution has to be considered as well. Although in our previous study no functional differences were found between term and preterm animals held in normoxia [13], no transcriptome analysis has been performed on term animals. Some of the expressional differences might be explained by prematurity alone without a clear hyperoxic cause. A final limitation is the use of selective filtering of the obtained results. First, filtering for small RPKM’s and fold changes may result in the loss of meaningful subtle dysregulated master switch molecules. The used software, IPA (Upstream Regulator Analysis and Network Generation Algorithm) is also dependent on prior knowledge and many expression-regulating relationships remain still to be discovered or described. This may bias our analysis, in favor of molecules which have been frequently investigated.

Despite these theoretical limitations, other results of our experiment show obvious similarities with previous studies analyzing specific molecules which may be involved in the pathophysiology of BPD.[27,48,49] The first two studies [27,48] examined the changes in expression profile during the last stages of lung development in rats without any hyperoxic lung damage. Important genes which were upregulated during the saccular stage were those involved in development. Both studies found the Wnt signaling pathway to be affected by different genes like Fzd1 (frizzled-1) or Ptn (pleiotrophin). Our data demonstrated DKK1, an inhibitor of Wnt signaling to be upregulated after exposure to hyperoxia. In the study of Bhattacharya [49] whole lung tissue was used from mice exposed to 100% hyperoxia for 10 days. They found Ahr to be a key regulator in the pathophysiology of hyperoxic lung damage. This receptor is known to activate CYP1A1, one of the main dysregulated molecules in our database. Furthermore, Cdkn1a (cyclin-dependent kinase inhibitor 1a), which is a cell cycle regulator, is upregulated both in mice as in our rabbit lung tissue. The exact mechanism how this gene is involved in the pathophysiology of hyperoxic lung injury remains to be determined.

To conclude, our study is the first to perform whole transcriptome analysis on hyperoxia-induced, preterm rabbit lung tissue identifying several central molecular mediators. The major pathophysiological features of BPD we identified are inflammation, lung developmental arrest, dysregulated vasculogenesis and increased ROS metabolism. We will use the molecular master switches revealed in this analysis for the future definition of novel preventive strategies to avoid or reduce the occurence of BPD.

## Supporting Information

S1 Table All dysregulated transcripts. FC: fold change, FDR: false discovery rate.
(XLSX)

S2 Table All activated and inhibited upstream regulators. FC: fold change, z-score and p-value of overlap.
(XLSX)

S3 Table Downstream effectors of TNFα. FC: fold change.
(XLSX)

S4 Table Downstream effectors of KLF2. FC: fold change.
(XLSX)

S5 Table Downstream effectors of NFE2L2. FC: fold change.
(XLSX)
